# A Comparative Study of Intense Pulsed Light with Two Different Filters in Meibomian Gland Dysfunction: A Prospective Randomized Study

**DOI:** 10.3390/jcm14010199

**Published:** 2025-01-01

**Authors:** Hayoung Lee, Yoo Young Jeon, Kyu Sang Eah, Nahyun Park, Yea Eun Lee, Jeewon Han, Chung Min Lee, Changmin Kim, Ho Seok Chung, Jae Yong Kim, Hun Lee

**Affiliations:** 1 Department of Ophthalmology, Asan Medical Center, University of Ulsan College of Medicine, Seoul 05505, Republic of Korea; glory2822@naver.com (H.L.); cheese_sauce@naver.com (Y.Y.J.); kseah0124@gmail.com (K.S.E.); laurenpark66@gmail.com (N.P.); yeaeun812@gmail.com (Y.E.L.); jenny4132@naver.com (J.H.); chungminlee1215@gmail.com (C.M.L.); kcm8821@naver.com (C.K.); chunghoseok@gmail.com (H.S.C.); jykim2311@amc.seoul.kr (J.Y.K.); 2 Department of Ophthalmology, Asan Medical Institute of Convergence Science and Technology (AMIST), University of Ulsan College of Medicine, Seoul 05505, Republic of Korea; 3 Department of Ophthalmology, Brain Korea 21 Project, University of Ulsan College of Medicine, Seoul 05505, Republic of Korea; 4 Center for Cell Therapy, Asan Medical Center, Seoul 05505, Republic of Korea

**Keywords:** acne filter, 590-nm filter, intense pulsed light, matrix metalloproteinase-9, meibomian gland dysfunction

## Abstract

**Objectives:** To compare the long-term efficacy and safety of intense pulsed light (IPL) treatments using a 590-nm and an acne filter. **Methods:** In this prospective, randomized, paired-eye trial study, 30 patients with moderate and severe meibomian gland dysfunction (MGD) were followed up for at least one month after their last treatment. Group A received IPL treatment with an acne filter, a type of notch filter that blocks wavelengths between 600 and 800 nm, allowing IPL to emit wavelengths between 400–600 nm and 800–1200 nm. Group B received treatment with a 590 nm filter, a type of cut-off filter that blocks wavelengths below 590 nm. Clinical parameters, including tear osmolarity, matrix metalloproteinase (MMP)-9 expression, tear break-up time, ocular surface staining scores, Schirmer’s test I, lid margin telangiectasia scores, MG expressibility/secretion scores, and Ocular Surface Disease Index scores, were measured at baseline, 1, 6, and 12 months after their last treatment. **Results:** In the linear mixed model, significant time effects on all clinical parameters, except for MMP-9 grades and Schirmer’s test I results, were observed within each group. However, interactions between time points (baseline, 6 months, 12 months) and groups (Group A, B) were not significant. The generalized estimating equation model showed no significant interaction between time points and groups for MMP-9 positivity; however, a significant time effect on MMP-9 positivity was observed in Group A, with a decrease at 12 months after their last treatment when compared to baseline and 6 months. **Conclusions:** The IPL treatment modality for moderate to severe MGD demonstrated a significant therapeutic effect for one year under strictly controlled self-administration of other treatments. IPL treatment using acne filter is a promising treatment option for reducing MMP-9 positivity in MGD patients.

## 1. Introduction

Meibomian gland dysfunction (MGD) is a chronic, diffuse abnormality of the meibomian glands, commonly characterized by terminal duct obstruction and qualitative or quantitative changes in glandular secretion [1]. Reduced meibum secretion or changes in its quality, such as increased viscosity, loss of omega-hydroxyl fatty acids, and alterations in the lipid profile, can increase tear film evaporation, leading to tear hyperosmolarity, ocular surface inflammation, cell apoptosis (including the loss of goblet cells that secrete mucins), and the clinical signs and symptoms of dry eye disease [2,3].

Traditional treatments for MGD include tear substitutes, topical cyclosporine, topical corticosteroids, oral antibiotics, omega-3 supplements, warm compresses, manual meibomian gland expression, intraductal probing, microblepharoexfoliation, and vectored thermal pulsation [4,5,6,7,8,9]. Although conventional treatments have proven to be effective, their effects are often short-term [10]. Alternatively, intense pulsed light (IPL), proposed as a novel treatment in 2015, has therapeutic effects that can last for more than six months [9,11,12]. IPL is a noncoherent, polychromatic light source with a broad wavelength spectrum (400–1200 nm), adjustable through proper filters [13]. Light is absorbed by chromophores, such as melanin, hemoglobin, and water, inducing selective photothermolysis. [9] The mechanisms through which IPL affects the meibomian gland include localized destruction of telangiectasia below the skin via thrombosis, photomodulation, heating and liquefying of the meibum, eradicating *Demodex folliculorum* mites, modulating the secretion of proinflammatory and anti-inflammatory molecules, suppressing matrix metalloproteinases (MMP), and altering reactive oxygen species levels [14].

Physicians can control penetration depth and selective chromophore targeting during IPL treatment by choosing specific filters, which are classified into cut-off and notch types. Cut-off filters, such as the 590-nm filter, block wavelengths below a certain threshold; while acne filters—a type of notch filter—block wavelengths between 600 and 800 nm, allowing the emission of 400–600 nm and 800–1200 nm wavelengths during IPL treatment [6,15,16]. With the 590-nm filter, a significant portion of energy is absorbed by melanin, which limits its ability to effectively target hemoglobin in both superficial and deeper vessels. By contrast, the acne filter minimizes melanin absorption by blocking the 600–800 nm range while effectively targeting hemoglobin in both the superficial (400–600 nm) and deeper (800–1200 nm) blood vessels [6,15,16]. Hemoglobin demonstrates its highest absorption coefficient at 400–600 nm, which declines sharply between 600 and 800 nm and stabilizes at a certain level beyond 800 nm [17]. Therefore, the shorter wavelength band (400–600 nm) targets porphyrins and hemoglobin in superficial vessels, while the longer band (800–1200 nm) targets hemoglobin in deeper vessels [6,15,16]. Light absorbed by hemoglobin is converted into heat, raising the vessel’s core temperature to 80–90 °C, which induces coagulation and thrombosis [12]. This process disrupts key reservoirs of inflammatory mediators, eliminating a primary source of inflammation in the meibomian glands and eyelids [12]. The dual-band targeting capability of the acne filter enhances its therapeutic efficacy for conditions like MGD by effectively targeting inflammation.

In this retrospective study, the authors demonstrated the efficacy and safety of IPL with an acne filter [6]. Additionally, in a prospective, randomized clinical trial, the authors found that IPL treatment using an acne filter yielded significant improvement in clinical signs, and its treatment effect was comparable to that of IPL treatment using a conventional filter [18]. However, these results were based on a short-term follow-up of one month after IPL treatment. Therefore, in this prospective, randomized, paired-eye study, the authors aim to compare the long-term efficacy and safety of IPL treatments using a 590-nm filter and an acne filter. The authors also investigated the effects of IPL treatment alone by restricting access to other treatments, such as warm compresses.

## 2. Materials and Methods

This prospective, randomized paired-eye study was approved by the International Review Board of Asan Medical Center and the University of Ulsan College of Medicine, Seoul, South Korea (2021-0815). This study adhered to the tenets of the Declaration of Helsinki and followed good clinical practice guidelines. Signed informed consent was obtained from all participants prior to enrollment.

Patients over 18 years of age with moderate to severe MGD and an Ocular Surface Disease Index (OSDI) score of 23 or higher were included in this study. Moderate to severe MGD was diagnosed when both symptoms (such as ocular discomfort, itching, or photophobia) and clinical signs were moderate to severe [19]. MGD clinical signs were considered moderate to severe in the following cases: decreased or cloudy/toothpaste-like secretions from the meibomian gland expressor, more than two obstructions of the meibomian gland orifices, or more than two definite telangiectasias in the lid margin. Exclusion criteria included the following: Sjögren’s syndrome; autoimmune disease; previous intraocular or ocular surgery; eyelid malposition; glaucoma with topical medication; eyelid malposition; ocular infection; non-dry eye ocular inflammation; ocular allergy; use of contact lenses during the study period; clinical skin treatments within two months prior to this study; semi-permanent makeup or tattoos; pigmented lesions at the treatment site; and pregnancy or lactation.

**Figure 1:**
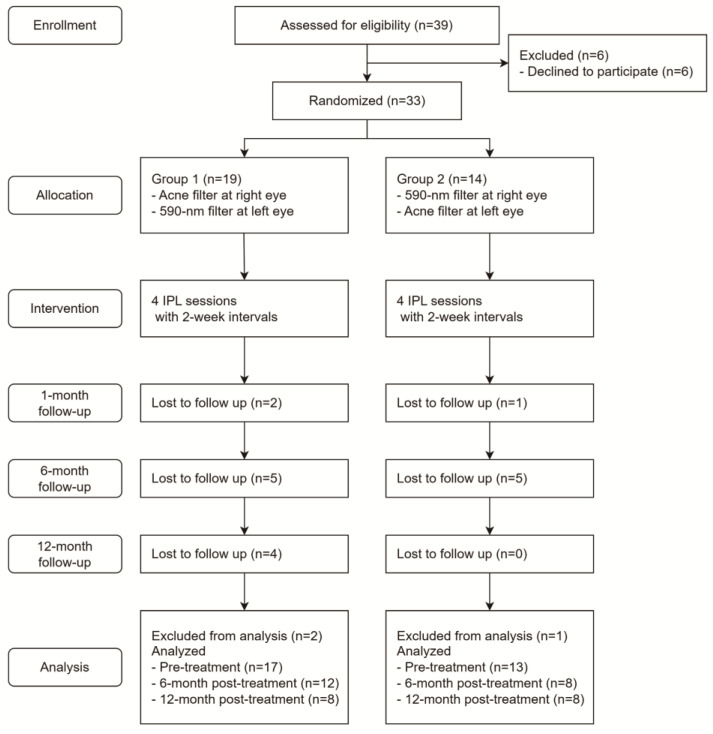
Flow diagram according to the Consolidated Standards of Reporting Trials (CONSORT) statement, showing enrollment, randomization, and patient flow in this study.

A total of 33 patients were randomly assigned and divided into two separate groups (Figure 1). A randomization sequence was created using random block sizes of 2, 4, and 6 by an independent doctor. The allocation sequence was concealed from the physician enrolling and assessing the patients using numbered, opaque, and sealed envelopes. Outcome assessors and data analysts were blinded to the allocations. In Group I, 19 patients received IPL treatments using an acne filter in the right eye and a 590-nm filter in the left eye. In Group II, 14 patients received IPL treatments using a 590-nm filter in the right eye and an acne filter in the left eye. One month after the fourth IPL treatment performed at two-week intervals, two patients in Group I and one patient in Group II were lost to follow-up. Six months after the final IPL treatment, an additional five patients in each group were lost to follow-up. Twelve months after the final treatment, an additional four patients in Group I were lost to follow-up. Patients who were not followed up one month after treatment were excluded from the analysis. The flow of the patients through this study is shown in Figure 1.

After a two-week washout period, all patients underwent four IPL treatment sessions at two-week intervals, administered by one physician (H.L.). Before the first treatment, Fitzpatrick skin typing was performed, and the IPL machine (M22 Optima IPL; Lumenis, Yokneam, Israel) was adjusted accordingly [20]. Topical anesthesia was instilled, and ultrasonic gel was applied to the treatment area. Both eyes were closed and covered with a metal spatula, starting with the right eye. For the lower eyelid, the margin was exposed by gently pulling it down with the IPL probe to maximize contact. Patients received approximately 13 light pulses from the right preauricular area, across the cheeks and nose, to the left preauricular area (two times; total 26 light pulses). For the upper eyelid treatment, a metal spatula was gently inserted between the bulbar conjunctiva and the upper eyelid. Patients received 2–3 light pulses after slightly tenting the metal spatula upwards to protect ocular structures. Immediately after IPL treatment, meibomian gland expression was performed on all four eyelids using expressor forceps, applying gentle pressure until the meibum was adequately expressed. Throughout the IPL treatment and the subsequent 12-month follow-up period, no other treatments except for 0.15% sodium hyaluronate (New Hyaluni, Taejoon, Seoul, Republic of Korea) were allowed, including warm compresses and lid scrubs.

Clinical parameters were measured before the first treatment, and at 1, 6, and 12 months after the final treatment. To minimize the impact of each test on subsequent results, tests were performed in the same order with at least five-minute intervals. The tests were conducted in the following order: tear osmolarity and extracellular MMP-9 measurements; biomicroscopic examination of tear film break-up time (TBUT) using fluorescein; ocular surface staining [assessed by National Eye Institute (NEI) scale, Sjögren’s International Clinical Collaborative Alliance (SICCA) Ocular Staining Score scale, and Oxford scale]; Schirmer’s test I without topical anesthesia; slit-lamp microscopy to examine lid margins [lid wiper epitheliopathy (LWE) and vascularity] and meibomian glands; and finally, the OSDI questionnaire.

MMP-9 levels were measured using an InflammaDry immunoassay (Rapid Pathogen Screening, Inc., Sarasota, FL, USA). A red line in the result zone along with a blue line in the control zone indicated a positive test result (MMP-9 ≥ 40 ng/mL). The vividness of the red line, which increased proportionally to the concentration of MMP-9, was used for semi-quantitative interpretation based on the manufacturer’s grading index (0 = none, 1 = trace, 2 = weak positive, 3 = positive, 4 = strong positive) [21]. For analysis, MMP-9 grades were further classified into two categories: positive and negative. Grades 2 through 4 were defined as positive for MMP-9, while grades 0 and 1 were defined as negative for MMP-9 [22]. Fluorescein staining of the lid wiper was graded from 0 to 3 for each of two characteristics: the linear area of involvement (0 = <2 mm; 1 = 2–4 mm; 2 = 5–9 mm; 3 = ≥10 mm) and the severity of staining (0 = absent; 1 = mild; 2 = moderate; 3 = severe). The total grade for fluorescein staining of the lid wiper was the average of the grades for the linear area and severity of staining (0 = <0.5; 1 = 0.5–1.0; 2 = 1.25–2.0; 3 = 2.25–3.0) [23]. Lid margin telangiectasias were assessed and scored for both upper and lower lids (0 = no or slight redness in the lid margin conjunctiva and no telangiectasia crossing MG orifices; 1 = redness in the lid margin conjunctiva and no telangiectasia crossing MG orifices; 2 = redness in the lid margin conjunctiva and telangiectasia crossing MG orifices with a distribution of less than half the full length of the lid; 3 = redness in the lid margin conjunctiva and telangiectasia crossing MG orifices with a distribution of half or more of the full length of the lid) [24]. MG expressibility scores were summed by scoring 0 or 1 based on meibum expression from each of the eight glands located in the center of each upper and lower eyelid. The secretion scores were calculated based on the expressibility and quality of the meibum in the eight glands in the center of each upper and lower eyelid. The quality of the meibum was scored from 0 to 3 (0 = clear fluid; 1 = cloudy fluid; 2 = cloudy fluid with particles; 3 = opaque, toothpaste-like meibum or solid obstruction with MG plugging) and these scores were summed across the eight glands to obtain a score ranging from 0 to 24 [25]. At every visit, intraocular pressure (IOP) and best corrected visual acuity (BCVA) were measured. Additionally, slit-lamp microscopic examination was performed to assess adverse events, such as loss of eyelashes, trichiasis, and iris tissue damage. Possible dermatologic adverse events, including redness, hyperpigmentation or hypopigmentation, blistering, and swelling, were also evaluated.

### Statistical Analysis

For the analysis, patients were reclassified into groups A and B based on the specific type of filter used during IPL treatments. Regardless of whether it was the right or left eye, Group A received IPL treatments with an acne filter, while Group B received treatments with a 590-nm filter. The primary outcome measures were intergroup comparisons of changes from baseline in upper and lower lid margin telangiectasia scores, MMP-9 grades and positivity rates, and TBUT at 6 and 12 months after the final treatment. The secondary outcome measures included intergroup comparisons of changes from baseline in MG secretion scores, ocular surface staining scores, and OSDI scores at 6 and 12 months after the final treatment. Statistical analysis was performed using SPSS software, version 23.0 (IBM, Armonk, NY, USA). Possible differences between the two groups and across the three-time points for clinical outcome measurements, except for MMP-9 positivity, were evaluated using a linear mixed model. For dichotomous variables, such as MMP-9 positivity, a generalized estimation equation model was used. Both models used an unstructured covariance matrix, and post hoc tests with Bonferroni correction for multiple comparisons were performed using three pairwise comparisons.

Patients were further classified into two subgroups based on changes in MG expressibility in the lower lid at six months after the final treatment: responding and non-responding subgroups. MG expressibility is a key baseline characteristic that correlates with ocular surface parameters and cytokines [26]. In the linear mixed model, no significant differences were observed in most parameters between the 6-month and 12-month results in both groups. Additionally, due to the higher number of missing values at 12 months compared to 6 months, the 6-month results were used for the subgroup analysis. Patients were classified as responding if their MG expressibility scores increased by more than three points compared to pre-IPL treatment scores. Mann–Whitney U tests and Fisher exact tests were performed to compare baseline clinical parameters between the responding and non-responding subgroups. *p* < 0.05 was considered statistically significant.

## 3. Results

**Figure 2:**
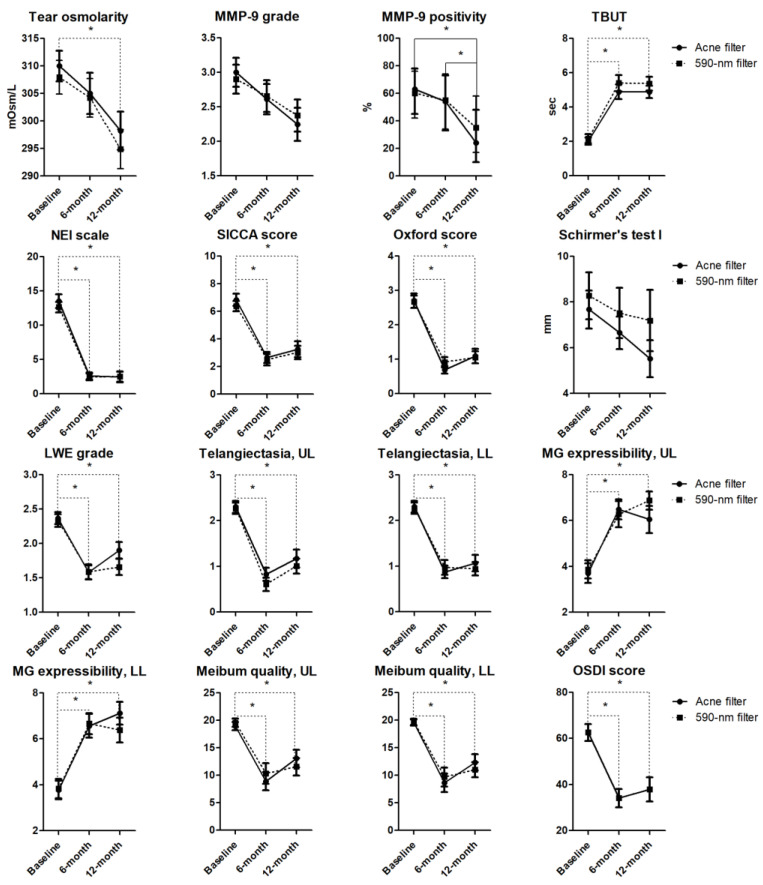
Long-term changes in clinical parameters, including tear osmolarity, matrix metalloproteinase-9 grade, matrix metalloproteinase-9 positivity, tear film break-up time, National Eye Institute scale, Sjögren’s International Clinical Collaborative Alliance score, Oxford score, Schirmer’s test I, lid wiper epitheliopathy grade, lid telangiectasia score, lid meibomian gland expressibility/secretion score, and Ocular Surface Disease Index score from baseline to 12 months after their last treatment. Points and error bars in the graphs indicate fixed effects estimates ± standard error (all except MMP-9 positivity). Points and error bars in graph C indicate estimated proportions and 95% confidence intervals (MMP-9 positivity). The dotted line was statistically significant in both Groups A and B, while the solid line was only significant in Group A. * *p* < 0.05 using a linear mixed model or generalized estimating equations model with post hoc tests and Bonferroni correction for multiple comparisons. (Top first) Acne filter: *p* = 0.023 (baseline vs. 12 months), 590-nm filter: *p* = 0.022 (baseline vs. 12 months); (Top third) Acne filter: *p* < 0.001 (baseline vs. 12 months), *p* = 0.006 (6 months vs. 12 months); (From top fourth to second row third) all *p* < 0.001; (Third row first) Acne filter: *p* < 0.001 (baseline vs. 6-months), *p* = 0.011 (baseline vs. 12 months), 590-nm filter: all *p* < 0.001; (Third row second and third) all *p* < 0.001; (Third row fourth) Acne filter: *p* = 0.001 (baseline vs. 6 months), *p* = 0.010 (baseline vs. 12 months), 590-nm filter: *p* = 0.007 (baseline vs. 6 months), *p* < 0.001 (baseline vs. 12 months); (Bottom first) Acne filter: *p* = 0.002 (baseline vs. 6 months), *p* < 0.001 (baseline vs. 12 months), 590-nm filter: *p* < 0.001 (baseline vs. 6 months), *p* = 0.002 (baseline vs. 12 months); (Bottom second) Acne filter: *p* < 0.001 (baseline vs. 6 months), *p* = 0.011 (baseline vs. 12 months), 590-nm filter: all *p* < 0.001; (Bottom third and fourth) all *p* < 0.001. MMP-9, matrix metalloproteinase-9; TBUT, tear film break-up time; NEI, National Eye Institute; SICCA, Sjögren’s International Clinical Collaborative Alliance; LWE, lid wiper epitheliopathy; MG, meibomian gland; OSDI, Ocular Surface Disease Index.

This study comprised 30 consecutive patients (60 eyes), including 6 males (12 eyes) and 24 females (48 eyes), with a mean age of 66.0 ± 8.8 years (range: 43–80). The results of the clinical parameters at baseline, and at 6 and 12 months after the final treatment in both groups, are shown in Table 1 and Figure 2. In the linear mixed model, no significant interactions were observed between time points (baseline, 6 months, and 12 months) and groups (Group A and B) on tear osmolarity, TBUT, NEI scales, SICCA scores, Oxford scores, LWE grades, upper and lower lid margin telangiectasia scores, upper and lower lid MG expressibility/secretion scores, and OSDI scores. However, the time effects on these parameters were significant in each group (tear osmolarity: *p* = 0.023 for Group A and *p* = 0.022 for Group B; upper lid MG expressibility: *p* = 0.001 for Group A and *p* < 0.001 for Group B; all other measurements: *p* < 0.001 for Group A and B). The post hoc analysis results were consistent in each group. Significant improvements were observed in all parameters, except for tear osmolarity at 6 and 12 months after their last treatment compared to baseline (*p*-values in Figure 2). However, no significant differences were observed in these parameters between 6 and 12 months after their last treatment. Tear osmolarity improved significantly only at 12 months after their last treatment when compared to that at baseline (*p* = 0.021 for Group A and *p* = 0.023 for Group B).

In both groups, neither the time effects on MMP-9 grades and Schirmer’s test I results, nor the interaction between time points and groups for these parameters, were statistically significant. In the generalized estimating equation model, although no significant interaction between time points and groups was observed for MMP-9 positivity, the time effect on MMP-9 positivity was significant in Group A only (*p* = 0.006). Positivity rates of MMP-9 significantly decreased at 12 months after their last treatment compared to baseline and at 6 months after their last treatment (*p* = 0.434 for baseline vs. 6 months, *p* < 0.001 for baseline vs. 12 months, and *p* = 0.006 for 6 months vs. 12 months comparisons).

When combining the results from both filters, the time effects on tear osmolarity, MMP-9 grades and positivity, TBUT, NEI scales, SICCA scores, Oxford scores, LWE grades, upper and lower lid margin telangiectasia scores, upper and lower lid MG expressibility/secretion scores, and OSDI scores were significant (tear osmolarity: *p* = 0.001; MMP-9 grades: *p* = 0.017; MMP-9 positivity: *p* = 0.003; all other measurements: *p* < 0.001). In the post hoc analysis, significant improvements in all these parameters, except for tear osmolarity and MMP-9 grades and positivity, were observed at both 6 and 12 months after their last treatment compared to baseline. Comparisons of the *p*-values at baseline vs. 6 months and baseline vs. 12 months are as follows: lower lid secretion scores (*p* < 0.001 and *p* = 0.001); all other measurements (*p* < 0.001). No significant difference was observed in these parameters between 6 and 12 months after their last treatment. Tear osmolarity and MMP-9 grades improved significantly only at 12 months after their last treatment compared to baseline (tear osmolarity: *p* = 0.001; MMP-9 grades: *p* = 0.013 for baseline vs. 12-month comparisons), and the positivity rates of MMP-9 decreased significantly at 12 months after their last treatment compared to baseline and 6 months after their last treatment (*p* < 0.001 for baseline vs. 6-month and *p* = 0.003 for 6-month vs. 12-month comparisons).

Subgroup analysis based on the improvement in MG expressibility showed that the average age was younger in the IPL-responding subgroup compared to the non-responding subgroup in both Group A (*p* = 0.001) and Group B (*p* = 0.004) (Table 2). In Group A, the responding subgroup had significantly lower upper and lower eyelid MG expressibility scores (*p* = 0.009 for upper eyelid and *p* = 0.015 for lower eyelid), indicating more severe MG orifice obstruction compared to the non-responding subgroup. Additionally, the lower lid meibum secretion score was significantly higher in the responding subgroup (*p* = 0.018), suggesting that the meibum in the lower eyelid had a more toothpaste-like consistency compared to the non-responding subgroup.

## 4. Discussion

In this prospective, randomized, paired-eye study, the authors compared the long-term effects of IPL treatments using an acne filter and a 590-nm filter in patients with moderate to severe MGD. The authors’ findings demonstrated that IPL treatments with either filter, followed by meibomian gland expression, significantly improved clinical symptoms and signs of MGD at 6 and 12 months after their last treatment, even without anti-inflammatory eye drops or eyelid management. No significant differences were observed in tear osmolarity, MMP-9 expression, TBUT, ocular surface staining scores, lid margin abnormality scores, meibomian gland parameters, or subjective symptom scores between the two filters. Interestingly, positivity rates of MMP-9 in Group A decreased significantly at 12 months after their last treatment compared to baseline and at 6 months after their last treatment. Additionally, regardless of the filter type, the responding subgroup tended to be younger than the non-responding subgroup. Within the acne filter group, the responding subgroup had worse upper and lower lid meibum expressibility, and lower lid meibum quality, when compared to the non-responding subgroup.

In a previous study comparing IPL treatments using a vascular filter and a 590-nm filter for MGD, both filters significantly improved dry eye, MGD parameters, and the OSDI scores, immediately after the fourth IPL treatment. However, no significant differences were observed between the two filters with respect to these parameters [27]. Similarly, in the authors’ previous study evaluating results one month after the fourth treatment, both the acne filter and the 590-nm filter demonstrated good therapeutic efficacy for MGD with no significant differences in clinical parameters, including TBUT, MMP-9 expression, MG parameters, and OSDI scores [18]. Despite expectations of improved efficacy in lid margin telangiectasia with the dual-band filter, no significant difference was found between the dual-band filters and conventional cut-off filters [18,27]. This result may be due to the reliance on slit-lamp examinations for current evaluations, suggesting that more advanced technologies are needed to effectively assess the impact of IPL with notch filters on lid margin telangiectasia.

In the current study, both IPL filters demonstrated good therapeutic efficacy with no significant differences in clinical parameters. However, although no statistically significant interaction was observed between time points and groups for MMP-9 positivity, the acne filter group showed greater improvements in MMP-9 positivity rate compared to the 590-nm filter group. Positivity rates of MMP-9 in Group A significantly decreased at 12 months after their last treatment compared to baseline and at 6 months after their last treatment. MMP-9 is known as a nonspecific, late-phase, inflammatory marker in patients with dry eyes [28,29,30]. Despite the use of the MMP-9 immunoassay kit, therapeutic effects on MMP-9 may not have been fully captured by current evaluation methods. An immunoassay kit has been developed to detect elevated tear MMP-9 levels, showing good diagnostic performance and correlation with dry eye severity [31,32,33]. However, the performance of lateral flow immunoassays, like InflammaDry, may be affected by sample volumes [18,34]. Further studies analyzing tear cytokines, including chemokine ligand, C-C motif chemokine, tumor necrosis factor, interferon, interleukin, and tissue inhibitor of metalloproteinase are required to more accurately compare treatment effects [35].

In the authors’ previous one-month study, the responding subgroup, regardless of filter type, showed worse baseline lower lid meibum expressibility and secretion scores compared to the non-responding subgroup [18]. Additionally, only the acne filter group exhibited worse baseline TBUT and upper lid meibum expressibility in the responding subgroup. In the current study, the authors found that the responding subgroup, regardless of filter type, comprised younger participants when compared to the non-responding subgroup, possibly due to reduced lid muscle function affecting therapeutic effects despite the improved meibum quality from IPL [36]. Notably, in the acne filter group, the responding subgroup had worse baseline upper and lower lid meibum expressibility and lower lid meibum quality. Therefore, aggravated MG status, such as poor meibum expressibility and worse lower lid meibum quality, may identify suitable candidates for IPL therapy with the acne filter.

Currently, most previous studies did not control for patients’ self-administration of eye drops, which could be an important confounding factor [37]. Similarly, recent studies that controlled for self-administration of eye drops did not control for self-administered eyelid management, such as warm compresses and lid scrubs [38]. In the current study, patients were instructed to only use artificial tears and to refrain from using warm compresses and lid scrubs. Thus, this study’s strengths lie in the precise evaluation of the therapeutic effect of IPL alone, with restrictions on other treatment options, such as warm compresses, enhanced eyelid hygiene, anti-inflammatory agents, antibiotics, or omega-3 fatty acid supplements [36]. Moreover, the pathogenesis of MGD primarily involves ductal epithelium hyperkeratinization and increased meibum viscosity due to various factors, such as aging, hormonal disturbances, medication toxicity, breakdown products of meibomian lipids, or contact lens wear [39]. By conducting a paired-eye study with restrictions on other treatment options, the authors were able to compare the therapeutic effects of two filters while minimizing the influences of the other contributing factors.

A dual-band filter, such as the acne filter, offers several advantages in terms of safety. Blocking the 600–800 nm range reduces light absorption by melanin, which is associated with risks, such as pain, eyelash loss, epidermal damage, and pigmentation issues; it also helps prevent ocular complications. like anterior uveitis, iris atrophy, pupillary defects, and secondary angle-closure glaucoma [40,41,42,43]. To ensure precise application and to minimize adverse effects on the eyelids, a metal spatula was used instead of the metal eye goggles or adhesive shields typically used in IPL treatment. No deterioration in best corrected visual acuity or fluctuations in intraocular pressure were observed during the follow-up period. Furthermore, no adverse effects, including iris damage, and no local reactions in the periorbital skin were observed.

This study has certain limitations. First, the sample size was relatively small, necessitating larger studies to assess the long-term effectiveness and safety of IPL treatments with the acne filter. Second, all patients were Korean with Fitzpatrick skin type 3; however, results may vary across different ethnicities, requiring studies in diverse groups. Nonetheless, to the authors’ knowledge, this may be the first prospective, randomized study comparing long-term IPL efficacy between cut-off and notch filters using a paired-eye design, controlling for various confounding factors.

## 5. Conclusions

The authors demonstrated a significant therapeutic effect of IPL on moderate to severe MGD for one year in the context of strictly controlled, self-administration of other treatment modalities, with no observed local or systemic adverse effects. Along with using IPL alone for the treatment of moderate to severe MGD, IPL treatment using the acne filter is a promising alternative for MGD treatment in terms of reduction in MMP-9 positivity.

## Figures and Tables

**Table 1:** Comparison of clinical parameters before and after intense pulsed light treatment for moderate to severe meibomian gland dysfunction between Group A and Group B.

	Group A (Acne Filter)				Group B (590-nm Filter)				*p* Value ^c^
	Before	6-Month	12-Month	*p* Value ^a^	Before	6-Month	12-Month	*p* Value ^b^	
Tear osmolarity (mOsm/L)	309.97 (2.76)	305.01 (3.73)	298.22 (3.45)	0.023	307.93 (3.06)	304.18 (3.49)	294.88 (3.57)	0.022	0.895
MMP-9 grade	3.00 (0.21)	2.61 (0.22)	2.24 (0.24)	0.006	2.90 (0.21)	2.65 (0.23)	2.37 (0.23)	0.149	0.848
MMP-9 positivity (%)	63 (45–78)	54 (33–73)	24 (10–48)	0.077	60 (42–76)	55 (34–74)	35 (17–58)	0.219	0.688
TBUT (s)	2.02 (0.21)	4.89 (0.43)	4.88 (0.36)	<0.001	2.16 (0.27)	5.39 (0.47)	5.37 (0.40)	<0.001	0.802
NEI scale	13.50 (0.98)	2.60 (0.49)	2.40 (0.77)	<0.001	12.63 (0.77)	2.43 (0.50)	2.50 (0.74)	<0.001	0.828
SICCA staining score	6.83 (0.43)	2.65 (0.39)	3.25 (0.59)	<0.001	6.37 (0.38)	2.50 (0.41)	3.01 (0.49)	<0.001	0.959
Oxford staining score	2.70 (0.21)	0.69 (0.11)	1.09 (0.21)	<0.001	2.67 (0.18)	0.91 (0.15)	1.05 (0.18)	<0.001	0.630
Schirmer’s test I (mm)	7.67 (0.84)	6.65 (0.71)	5.52 (0.81)	0.071	8.27 (1.03)	7.51 (1.10)	7.19 (1.34)	0.652	0.816
LWE staining grade	2.37 (0.09)	1.58 (0.10)	1.90 (0.12)	<0.001	2.33 (0.09)	1.59 (0.11)	1.66 (0.12)	<0.001	0.744
Telangiectasia score									
Upper lid	2.30 (0.13)	0.82 (0.14)	1.17 (0.19)	<0.001	2.27 (0.13)	0.61 (0.15)	1.01 (0.16)	<0.001	0.791
Lower lid	2.30 (0.13)	0.87 (0.14)	1.06 (0.18)	<0.001	2.27 (0.13)	0.97 (0.16)	0.94 (0.15)	<0.001	0.815
Expressibility									
Upper lid	3.70 (0.43)	6.48 (0.44)	6.04 (0.59)	0.001	3.87 (0.40)	6.27 (0.57)	6.87 (0.40)	<0.001	0.628
Lower lid	3.77 (0.41)	6.57 (0.52)	7.11 (0.49)	<0.001	3.83 (0.42)	6.66 (0.46)	6.38 (0.54)	<0.001	0.849
Meibum quality									
Upper lid	18.93 (0.77)	8.90 (1.63)	13.03 (1.61)	<0.001	19.53 (0.75)	10.32 (1.87)	11.53 (1.58)	<0.001	0.564
Lower lid	19.53 (0.57)	8.62 (1.68)	12.34 (1.46)	<0.001	19.70 (0.57)	9.67 (1.72)	10.99 (1.39)	<0.001	0.872
OSDI score	62.56 (3.70)	34.10 (3.98)	37.86 (5.29)	<0.001	62.56 (3.70)	34.10 (3.98)	37.86 (5.29)	<0.001	0.872

Results are presented as fixed effects estimates (standard error) or estimated proportions (95% confidence intervals) and *p*-values from a linear mixed model or a generalized estimating equations model. ^a^ The *p*-value for the time effect in the Type III fixed effects test or model effects test when analyzing Group A separately. ^b^ The *p*-value for the time effect in the Type III fixed effects test or model effects test when analyzing Group B separately. ^c^ The *p*-value for the interaction in the Type III fixed effects test or model effects test when analyzing Group A and Group B together. MMP-9, matrix metalloproteinase-9; TBUT, tear film break-up time; NEI, National Eye Institute; SICCA, Sjögren’s International Clinical Collaborative Alliance; LWE, lid wiper epitheliopathy; OSDI, Ocular Surface Disease Index.

**Table 2:** Comparison of clinical parameters between acne and 590-nm filters in the responding and non-responding groups.

Parameters	Filter	Responding Group	Non-Responding Group	*p* Value
n (%)	Acne filter	12 (57.1%)	9 (42.9%)	□
	590-nm filter	11 (52.4%)	10 (47.6%)	□
Sex	Acne filter	4/8 (M/F)	1/8 (M/F)	0.338
	590-nm filter	3/8 (M/F)	2/8 (M/F)	>0.999
Age	Acne filter	61.00 (58.50–70.00)	73.50 (67.25–78.50)	0.001
	590-nm filter	64.00 (58.50–73.00)	71.00 (66.25–78.50)	0.004
Tear osmolarity (mOsm/L)	Acne filter	310.00 (300.00–320.00)	308.00 (290.25–322.50)	0.862
	590-nm filter	309.00 (297.50–330.00)	313.50 (287.00–324.50)	0.223
MMP-9 grade	Acne filter	3.00 (2.00–4.00)	3.00 (2.25–3.75)	0.862
	590-nm filter	2.00 (1.50–4.00)	3.00 (2.00–3.75)	0.173
MMP-9 positivity (%)	Acne filter	58.3%	66.7%	>0.999
	590-nm filter	36.4%	70.0%	0.198
TBUT (s)	Acne filter	1.00 (1.00–2.00)	2.50 (1.00–3.75)	0.169
	590-nm filter	1.00 (1.00–3.00)	2.00 (1.00–3.75)	0.557
NEI scale	Acne filter	16.00 (15.00–17.50)	11.00 (13.00–15.00)	0.082
	590-nm filter	14.00 (9.50–16.00)	11.00 (12.00–16.00)	0.973
SICCA staining score	Acne filter	8.00 (5.00–8.50)	6.50 (4.25–8.00)	0.277
	590-nm filter	6.00 (4.00–8.00)	6.50 (4.25–7.00)	0.863
Oxford staining score	Acne filter	2.00 (2.00–3.00)	3.00 (2.25–4.00)	0.508
	590-nm filter	3.00 (1.50–3.00)	3.00 (2.25–3.00)	0.114
Schirmer’s test I (mm)	Acne filter	8.00 (5.50–11.00)	5.00 (3.00–8.75)	0.382
	590-nm filter	8.00 (4.50–12.00)	5.50 (3.50–7.75)	0.973
LWE staining grade	Acne filter	2.00 (2.00–3.00)	2.00 (2.00–3.00)	0.554
	590-nm filter	2.00 (2.00–3.00)	2.00 (2.00–2.50)	0.468
Upper lid margin telangiectasia	Acne filter	2.00 (2.00–3.00)	2.00 (1.25–3.00)	0.422
	590-nm filter	3.00 (2.00–3.00)	2.00 (1.25–2.75)	0.705
Lower lid margin telangiectasia	Acne filter	3.00 (2.00–3.00)	2.00 (1.25–3.00)	0.277
	590-nm filter	2.00 (2.00–3.00)	2.00 (1.25–3.00)	0.863
Upper lid meibum expressibility	Acne filter	2.00 (1.00–3.00)	4.00 (2.50–6.75)	0.009
	590-nm filter	2.00 (2.00–3.50)	4.00 (2.50–6.00)	0.512
Lower lid meibum expressibility	Acne filter	3.00 (2.00–3.50)	6.00 (2.50–6.00)	0.015
	590-nm filter	3.00 (2.00–4.00)	6.00 (1.75–6.75)	0.072
Upper lid meibum quality	Acne filter	22.00 (17.00–22.50)	19.00 (17.25–20.00)	0.069
	590-nm filter	22.00 (20.00–24.00)	20.00 (16.50–20.00)	0.426
Lower lid meibum quality	Acne filter	21.00 (19.00–22.00)	18.00 (15.50–20.00)	0.018
	590-nm filter	21.00 (19.50–22.00)	17.50 (15.25–21.75)	0.251
OSDI score	Acne filter	65.90 (37.90–73.60)	69.85 (39.10–86.88)	0.808
	590-nm filter	68.20 (47.25–75.00)	57.50 (36.30–72.58)	0.512

Results are presented as median (interquartile range) or frequency, along with the exact significance probabilities (*p*-values) from the Mann–Whitney U test or Fisher’s exact test. MMP-9, matrix metalloproteinase-9; TBUT, tear film break-up time; NEI, National Eye Institute; SICCA, Sjögren’s International Clinical Collaborative Alliance; LWE, lid wiper epitheliopathy; OSDI, Ocular Surface Disease Index. □: No *p*-value is available.

## Data Availability

The datasets generated during and/or analyzed during the current study are available from the corresponding author on reasonable request.
